# Navigating Ankle Fracture Surgery in the Shadow of COVID-19

**DOI:** 10.1177/19386400241274539

**Published:** 2024-09-20

**Authors:** Justin P. Chan, Henry Hoang, Amanda Anderson, Andrew R. Hsu

**Affiliations:** The Department of Orthopaedic Surgery, University of California Irvine, Orange, California; The Department of Orthopaedic Surgery, University of California Irvine, Orange, California; The Department of Orthopaedic Surgery, University of California Irvine, Orange, California; The Department of Orthopaedic Surgery, University of California Irvine, Orange, California

**Keywords:** COVID-19, ankle fracture, outcomes, postoperative complications, peri-operative

## Abstract

**Background:**

This case control study aimed to evaluate the impact of preoperative COVID-19 diagnosis on postoperative complications in patients undergoing ankle fracture surgery using the National COVID-19 Cohort Collaborative (N3C) database. The investigation focused on the interplay between COVID-19 diagnosis timing, patient characteristics, and clinical outcomes, particularly considering the potential mechanisms by which COVID-19 may contribute to increased complications.

**Methods:**

This case control study included patients who underwent ankle fracture surgery using the N3C database. The cohort was divided into two groups: patients without COVID-19 diagnosis within 12 weeks of surgery (n = 16 806) and those with a positive COVID-19 diagnosis within 12 weeks of surgery (n = 369). Demographic factors were analyzed. Clinical outcomes encompassed deep vein thrombosis (DVT), sepsis, surgical site infection, bleeding, acute kidney injury, 30-day mortality, and 365-day mortality. Multivariate logistic regression analyses were conducted.

**Results:**

The COVID-19–positive cohort displayed a slightly higher mean age (52.95 ± 18.43 vs 51.62 ± 18.36, P = .169) and body mass index (34.88 ± 9.99 vs 33.86 ± 8.80, P = .028) compared to the negative cohort. Although some outcomes, such as DVT and sepsis, demonstrated slightly higher frequencies in the COVID-19–positive group, these differences were not statistically significant. Adjusted odds ratios (AORs) for various COVID-19 diagnosis periods were generally not significant, except for a heightened risk of 30-day all-cause mortality associated with COVID-19 positivity within 0 to 2 weeks of surgery (AOR = 6.29, P = .003).

**Conclusions:**

Preoperative COVID-19 diagnosis within 12 weeks did not exhibit a significant association with most postoperative complications. While this study did not unveil substantial COVID-19–related effects, acknowledging the broader context of the pandemic remains essential in guiding comprehensive patient care strategies.

**Level of Evidence:**

Level III

“Whether the preoperative COVID-19 diagnosis exhibits a significant association with most postoperative complications in patients undergoing ankle fracture surgery.”

## Introduction

COVID-19, caused by the severe acute respiratory syndrome coronavirus 2 (SARS-CoV-2) virus, has resulted in over 700 million confirmed cases worldwide.^1^ In addition to its well-known symptoms, such as respiratory issues and organ dysfunction, COVID-19 can also lead to thrombotic complications. Evidence suggests a high incidence of blood clotting in infected patients, with rates of deep vein thrombosis (DVT) reaching up to 79% shortly after intensive care unit admission and postmortem studies reporting thrombosis in 81% of cases.^2^ Interestingly, research indicates that early diagnosis of COVID-19 prior to scheduled surgery increases the risk of postoperative complications.^3^ To our knowledge, research of foot and ankle surgeries in relation to patients with COVID-19 is limited. Large-scale studies involving complications regarding postoperative complications for patients undergoing ankle surgeries during the COVID-19 pandemic are needed.

One common indication for foot and ankle surgery is an ankle fracture.^4^ Ankle fractures can result from trauma or high-impact injuries, such as falls or sports-related accidents. Without proper surgical intervention, these fractures may lead to long-term complications, including chronic pain, instability, or deformity. One of the most common complications following surgery includes infection. In addition, other complications include blood clots, nerve or blood vessel injury, ankle stiffness or instability, malunion, nonunion, and anesthesia-related complications.^5^ There have been limited studies evaluating how COVID-19 affects these complications following ankle surgeries.

The COVID-19 pandemic has had a significant impact on both elective and non-elective ankle surgeries. Elective ankle surgeries have been heavily disrupted due to resource allocation challenges, the risk of infection, shortages of personal protective equipment (PPE), and staffing constraints.^6^ Hospitals and health care systems have redirected resources to prioritize COVID-19 patients, leading to limited capacity for elective surgeries. In addition, the risk of COVID-19 transmission during hospital visits has prompted many health care facilities to postpone or cancel non-essential surgeries, including elective ankle procedures.^7^ On the other hand, non-elective ankle surgeries generally continued but with additional precautions. These include preoperative COVID-19 testing, enhanced infection control measures, and the allocation of resources based on urgency. The aim has been to balance the urgent health care needs of patients while prioritizing safety during the pandemic. There has not been a consensus within the international foot and ankle community on the complications and protocols regarding COVID-19 pandemic and both elective and non-elective ankle surgeries.^8^

Having a COVID-19 infection can significantly increase the risk of negative outcomes in patients undergoing ankle surgery. This is due to several biological mechanisms and potential complications associated with COVID-19. First, COVID-19 is known to cause a hypercoagulable state, increasing the risk of blood clots.^9^ This can lead to DVT or pulmonary embolism, which can be exacerbated by the immobility that follows surgery. The virus can directly affect the vascular endothelium, leading to endothelial dysfunction and increased clotting tendency. Another complication is respiratory compromise. The virus primarily affects the respiratory system, causing lung inflammation and impairing gas exchange. Patients with COVID-19 may have reduced lung function and compromised respiratory reserve, making them more susceptible to postoperative respiratory complications such as pneumonia or acute respiratory distress syndrome.^10^ The presence of respiratory symptoms or pre-existing lung damage can further worsen outcomes following ankle surgery.

Furthermore, COVID-19 can weaken the immune system, impair wound healing, and increase the risk of infection. The virus suppresses immune responses, making patients more susceptible to surgical site infections and other complications.^2^ The combination of a compromised immune system, surgical trauma, and potential exposure to the virus in health care settings can increase the risk of postoperative infections.

In addition, COVID-19 can lead to multiorgan dysfunction. The virus affects various organs beyond the respiratory system, including the cardiovascular system, kidneys, liver, and gastrointestinal tract.^11^ Patients with pre-existing organ dysfunction or those experiencing COVID-19–related organ damage may have increased vulnerability to surgical stress and postoperative complications.

We specifically examined whether a closer diagnosis of active COVID-19 infection to the operative date was associated with a higher incidence of postoperative complications, including DVT, sepsis, acute kidney injury (AKI), and mortality.

Our study hypothesized that patients testing positive for COVID-19 closer to their ankle surgery date would have a higher incidence of postoperative complications compared to those testing positive further in advance. In addition, we hypothesized that patients with a COVID-19 infection detected well before their scheduled surgery would be less likely to experience postoperative complications.

## Materials and Methods

The National COVID-19 Cohort Collaborative (N3C) is a research initiative that was established by the National Institutes of Health to address critical research questions related to the COVID-19 pandemic. It brought together stakeholders to create a centralized data platform that integrated electronic health records from multiple institutions, providing access to extensive clinical and research data on COVID-19 patients.

We conducted a case control study using data from the N3C, which comprises data collected from March 15, 2020 to April 2023 from 80 major academic and community institutions. International Classification of Diseases (ICD)-10 code U07.1 was used to identify COVID-19 positivity. This study included participants of all ages, and the COVID-19–positive cohort was compared with COVID-19–negative controls based on age, sex, race, diabetes, and hypertension. Comorbidity data were collected using ICD-10 and ICD-9 codes mapped to systematized nomenclature of medicine clinical terms (SNOMED CT) codes. Rows with missing demographic data or outcome data were excluded.

### Querying Process

Patients who underwent ankle surgery procedures were identified by utilizing specific Current Procedural Terminology codes, including 23491, 23616, 24498, 27187, 27125, 27766, 27769, 27792, 27814, 27822, 27823, and 27829. Patients in the COVID-19–positive group were included if they had a positive COVID-19 diagnosis within 90 days before their ankle surgery. This cohort was further stratified based on the timing of their COVID-19 diagnosis: 0 to 2 weeks before ankle fracture surgery, 2 to 6 weeks before surgery, and 6 to 12 weeks before surgery. After identifying the COVID-19–positive and COVID-19–negative cohorts who underwent ankle surgery, we used ICD-10 codes to query for conditions such as pulmonary thrombosis, sepsis, AKI, surgical site infection, and death within 30 days of surgery. We selected patients who developed these respective complications within 0 to 84 days after their surgery. Comorbidity data were collected from ICD-10 codes mapped to SNOMED CT codes. Rows with missing data were omitted from the final analysis.

### Analysis Variables

The primary independent variable in our study was COVID-19 positivity, which we defined as having a positive COVID-19 diagnosis 0 to 90 days before ankle surgery. Additional patient characteristics of interest included age, race, gender, body mass index (BMI), smoking status, diabetes, and hypertension. Our primary outcomes were 30-day mortality, 365-day mortality, venous thromboembolic events, sepsis, AKI, and surgical site infection.

### Statistical Analysis

We used descriptive statistics to evaluate baseline patient characteristics. Continuous variables were expressed as mean ± standard deviation. Categorical variables were compared between groups using odds ratios. We considered *P*-values less than .05 to be statistically significant. We created two multivariate regression models. The first regression model was used to determine which predictor variables were significantly associated with our primary outcome. The second regression model was completed with only significant predictor models included to generate adjusted odds ratios (AORs).

## Results

**Figure 1.**
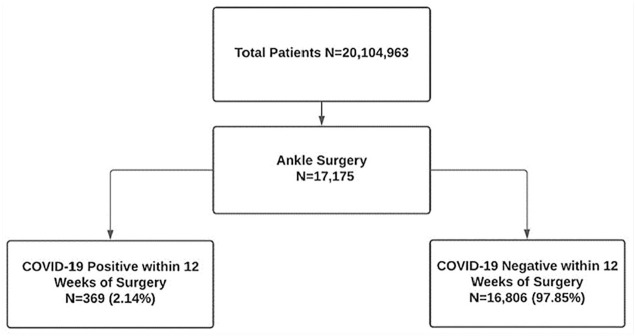
Flowchart schematic of study querying method.

Out of 20 104 963 patients queried, 17 175 individuals underwent surgery related to ankle injury. The study groups were comprised of patients who were negative for COVID-19 within 12 weeks of their surgery (97.8%) and patients who were COVID-19 positive within 12 weeks of their surgery (2.14%; Figure 1). Age (51.6 ± 18.4 vs 52.9 ± 18.43, *P* = .169), sex (F 60.5% vs F 65.0%, *P* = .078), and Charlson Comorbidity Index (CCI) scores (1.87 ± 2.70 vs 2.01 ± 2.54, *P* = .315) were comparable between the two groups. Body mass index (33.86 ± 8.80 vs 34.88 ± 9.99, *P* = .028) differed between the two groups as the COVID-19 positive within 12 weeks of surgery group had higher BMIs (Table 1). Queried postoperative complications included DVT, sepsis, surgical site infection, bleeding, AKI, 30-day all-cause mortality, and 365-day all-cause mortality (Table 2). All data categories with less than 20 counts were denoted as <20 in accordance with N3C policy.

**Table 1.** Comparison of Patient Demographics and Medical Comorbidities Between COVID-19–Positive and COVID-19–Negative Cohorts.

Characteristic	No COVID-19 diagnosis within 12 weeks of surgery (*n* = 19 973)	COVID-19 diagnosis within 12 weeks of surgery (*n* = 369)	*P*
Mean age, years (SD)	51.62 (18.36)	52.95 (18.43)	.169
Sex, %			.078
Male	39.49%	34.96%	
Female	60.51%	65.04%	
Race, *n* (%)			.730
White	12,777 (76.0)	276 (74.8)	
Black or African American	3547 (21.2)	86 (23.3)	
Asian	319 (1.89)	<20	
Native Hawaiian or other Pacific Islander	92 (0.54)	<20	
American Indian or Alaska Native	71 (0.42)	<20	
BMI, kg/m^2^ mean (SD)	33.86 (8.80)	34.88 (9.99)	.028
Charlson Comorbidity Index, mean (SD)	1.87 (2.70)	2.01 (2.54)	.315

Abbreviations: SD, standard deviation; BMI, body mass index.

**Table 2.** Comparison of Perioperative Complication Rates Between COVID-19–Positive and COVID-19–Negative Cohorts.

Clinical outcome	No COVID-19 diagnosis within 12 weeks of surgery (*n* = 16 806)	COVID-19 diagnosis within 12 weeks of surgery (*n* = 369)	*P*
Venous thromboembolism^a^	197 (1.17)	<20	.803
Sepsis^a^	150 (0.89)	<20	.392
Surgical site infection^a^	432 (2.57)	<20	.867
30-day mortality	32 (0.19)	<20	.038
365-day mortality	232 (1.38)	<20	.180

Values are reported as *n* (%) unless otherwise specified.

a Clinical outcomes occurring within 90 days of surgery.

**Figure 2.**
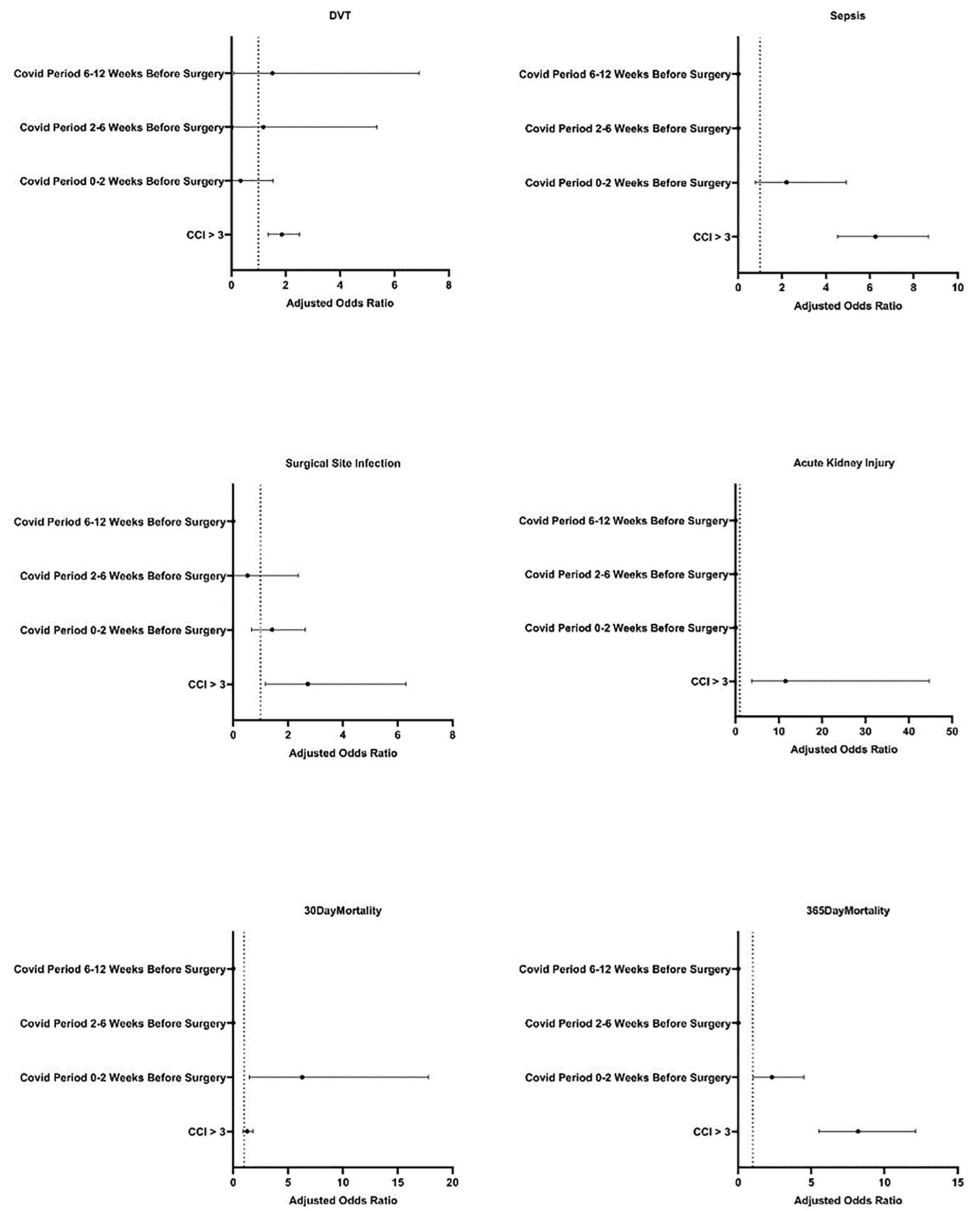
Odds ratios for postoperative complications in patients with a confirmed COVID-19 diagnosis compared to controls without a positive COVID-19 test for the 84 days prior to surgery. Error bars represent 95% CI. Abbreviation: CCI, Charlson Comorbidity Index.

On multivariate logistic regression, COVID-19 positivity within 0 to 2 weeks of surgery, and CCI score >3 were found to be significant predictors of 30-day mortality. After adjusting for COVID-19 positivity within 0 to 2 weeks of surgery and CCI >3 score, COVID-19 positivity within 0 to 2 weeks before surgery had the following AORs for DVT, sepsis, surgical site infection, 30-day all-cause mortality, and 365-day all-cause mortality: 0.34 (95% CI [0.02, 1.54]), 2.20 (95% CI [0.77, 4.93]), 1.42 (95% CI [0.67, 2.63]), 6.29 (95% CI [1.50, 17.81]), and 2.30 (95% CI [1.02, 4.48). CCI >3 had the following AORs for DVT, sepsis, surgical site infection, 30-day all-cause mortality, and 365-day all-cause mortality: 1.86 (95% CI [1.36, 2.50]), 6.26 (95% CI [4.54, 8.69]), 2,72 (95% CI [1.18, 6.30]), 1.28 (95% CI [0.91, 1.80]), and 8.20 (95% CI [5.53, 12.15]; Table 3, Figure 2). No odds ratios were reported for bleeding and AKI because not enough cases occurred for model fit.

**Table 3.** Association Between COVID-19 and Perioperative Complication Risk.

DVT			
Characteristic	Odds ratio	Adjusted odds ratio	*P*
CCI >3	1.50 [1.06, 2.10]	1.86 [1.36, 2.50]	<.001
COVID period 0-2 weeks before surgery	0.34 [0.02, 1.52]	0.34 [0.02, 1.54]	
COVID period 2-6 weeks before surgery	1.19 [0.07, 5.41]	1.18 [0.07, 5.36]	
COVID period 6-12 weeks before surgery	1.44 [0.08, 6.61]	1.51 [0.09, 6.92]	
Sepsis			
Characteristic	Odds ratio	Adjusted odds ratio	*P*
CCI >3	3.85 [2.67, 5.58]	6.26 [4.54, 8.69]	<.001
COVID period 0-2 weeks before surgery	2.11 [0.73, 4.75]	2.20 [0.77, 4.93]	.288
COVID period 2-6 weeks before surgery	0.00 [0.00, 0.00]	0.00 [0.00, 0.00]	.872
COVID period 6-12 weeks before surgery	0.00 [0.00, 0.00]	0.00 [0.00, 0.00]	.684
Surgical site infection			
Characteristic	Odds ratio	Adjusted odds ratio	*P*
CCI > 3	2.24 [1.79, 2.80]	2.72 [1.18, 6.30]	<.001
COVID period 0-2 weeks before surgery	1.37 [0.65, 2.55]	1.42 [0.67, 2.63]	.089
COVID period 2-6 weeks before surgery	0.54 [0.03, 2.43]	0.52 [0.03, 2.38]	.977
COVID period 6-12 weeks before surgery	0.00 [0.00, 0.01]	0.00 [0.00, 0.01]	.980
Acute kidney injury			
Characteristic	Odds ratio	Adjusted odds ratio	*P*
CCI >3	11.53 [3.74, 44.67]	17.52 [6.48, 47.29]	<.001
COVID period 0-2 weeks before surgery	0.00 [0.00, 0.00]	0.00 [0.00, 0.00]	
COVID period 2-6 weeks before surgery	0.00 [0.00, 0.00]	0.00 [0.00, 0.00]	
COVID period 6-12 weeks before surgery	0.00 [0.00, 0.00]	0.00 [0.00, 0.00]	
30-day all-cause mortality			
Characteristic	Odds ratio	Adjusted odds ratio	*P*
CCI >3	1.69 [0.82, 3.51]	1.28 [0.91, 1.80]	.155
COVID period 0-2 weeks before surgery	5.64 [1.34, 16.18]	6.29 [1.50, 17.81]	.003
COVID period 2-6 weeks before surgery	0.00 [0.00, 0.00]	0.00 [0.00, 0.00]	.987
COVID period 6-12 weeks before surgery	0.00 [0.00, 0.00]	0.00 [0.00, 0.00]	.988
365-day all-cause mortality			
Characteristic	Odds ratio	Adjusted odds ratio	*P*
CCI >3	4.22 [3.14, 5.72]	8.20 [5.53, 12.15]	<.001
COVID period 0-2 weeks before surgery	2.04 [0.90, 4.03]	2.30 [1.02, 4.48]	.026
COVID period 2-6 weeks before surgery	0.00 [0.00, 0.00]	0.00 [0.00, 0.00]	.976
COVID period 6-12 weeks before surgery	0.00 [0.00, 0.00]	0.00 [0.00, 0.00]	.979

Values are reported as odds ratio (95% CI). Abbreviations: DVT, deep vein thrombosis; CCI, Charlson Comorbidity Index.

## Discussion

The COVID-19 pandemic, instigated by the SARS-CoV-2 virus, ushered in unparalleled challenges for ensuring the safety of patients undergoing surgeries, including those for ankle fractures.^12^ While various measures such as PPE, patient isolation, meticulous patient transportation, and operating room sanitization were swiftly adopted, understanding the outcomes for patients with active COVID-19 infections undergoing surgery remains pivotal.

The N3C has been used to investigate orthopaedic procedures in the setting of COVID-19. Levitt et al^3^ investigated patients who underwent hip fracture surgeries and discovered a striking 30-day mortality rate of 14.6% for those who had surgery within a 7-day window before or up to 30 days after a COVID-19 diagnosis. In contrast, the mortality rate was only 3.8% for those without a COVID-19 diagnosis. Our findings align with these results, as we observed a heightened 30-day mortality rate of 0.56% in ankle fracture patients who had surgery within 0 to 2 weeks post–COVID-19 diagnosis compared to a 0.19% baseline mortality rate. It is worth noting that Levitt et al focused on data from March to December 2020, while our research spans over 2 years, from September 2020 to March 2023, and factors in patients who received COVID-19 vaccinations.

Another N3C cohort-based study, Pitts et al, explored outcomes for patients undergoing ankle fracture fixation.^13^They found heightened 30-day mortality rates for patients who had surgery either within the 7 days preceding or 30 days post a COVID-19 inpatient hospitalization. Interestingly, there was not any discernible difference in postoperative complication profiles. This may be attributed to the relatively non-invasive nature of ankle fracture fixations.

A retrospective study by Bryant et al which investigated the temporal effects of COVID-19 on postoperative outcomes across various surgical specialties demonstrated a pattern suggesting that for every 10-day delay in surgery post–COVID-19 diagnosis, there was an approximate 1% reduction in the risk of postoperative cardiovascular complications, such as DVT, pulmonary embolism, stroke, and myocardial infarction.^14^ This contrasts with our findings, where we did not observe any significant increase in complication rates 1 month after a COVID-19 diagnosis. Interestingly, the time-dependent effect in Bryant et al’s study extended throughout their observation period of up to 600 days post-infection. Overall, the inclusion of multispecialty data and persistence of COVID-19 effects over their 600-day observation period make it challenging to determine an ideal surgical timeline for ankle surgery following a COVID-19 infection. Similarly, a retrospective cohort study by Forlenza et al investigated the temporal effects of COVID-19 infection and postoperative complication profiles in hip and knee arthroplasty.^15^ Forlenza et al demonstrated a notable decline in the odds ratios of thromboembolic events, cardiopulmonary complications, renal injury, and urinary tract infections along with increased time following COVID-19 infection; however, patients are still at higher risk of thromboembolic events up to 3 months. The contrasting elements may be due to the study’s observation dates of January 2018 to April 2020, a period where COVID-19 vaccination was widely unavailable and the nature of hip and knee arthroplasty procedures being more invasive than ankle fracture fixation.

Finally, in our prior research utilizing the N3C cohort to examine the impact of COVID-19 infection timing on lumbar spinal fusions, we found that patients who underwent surgery within 2 weeks of a COVID-19 diagnosis exhibited a heightened risk of thromboembolic events and sepsis.^16^ In this case, there was a clear temporal association with COVID-19 and postoperative complication profiles. The contrasting elements are likely explained by the fact that ankle fracture fixation is inherently less invasive procedure than spinal lumbar fusions. Taken together, this suggests that COVID-19 can exert different temporal effects depending on the invasiveness of the procedure.

Ankle fractures are urgent medical conditions necessitating immediate surgical intervention. Delaying surgical treatment based on a minimal risk of 30-day mortality is not a viable strategy. Delayed intervention for closed ankle fractures has been associated with a significant rise in morbidity including nonunion, malunion, and post-traumatic arthritis.^13^ The complications of delaying are exacerbated further in the setting of open ankle fractures.^17^ Instead, a more nuanced approach is required during the peri-operative phase. Enhanced preoperative evaluations, encompassing a thorough review of a patient’s recent medical history, can significantly improve outcomes.

These findings are useful in highlighting the postoperative sequelae of COVID-19–positive patients who underwent ankle surgery. Nevertheless, our study has its limitations. The scope was restricted to ankle fractures, which precluded a deeper dive into other influential orthopaedic parameters. The N3C database does not contain information such as the severity of ankle fractures, the degree of comminution, radiographic parameters, and involvement of neurovascular structures. As such, complex ankle surgeries entail comprehensive tissue manipulation which can amplify the risk of complications like nerve damage or hindered healing due to compromised blood circulation.^18^ The intricate nature of these surgeries combined with the gravity of the injury could skew the relationship between the timing of a COVID-19 diagnosis and surgical outcomes.

Moreover, our study did not factor in the severity of the COVID-19 infection which can considerably influence postoperative complications. Severe COVID-19 cases, marked by respiratory distress or prolonged hospital stays, can undeniably affect the patient’s recovery trajectory post-surgery.^19^ It is entirely possible that severe cases of COVID-19 could lead to greater risk of postoperative complications, which warrants further study.^20^

In the context of ankle fracture surgery, a preoperative COVID-19 diagnosis was associated with increased 30-day mortality in patients who underwent surgery within 2 weeks of a positive COVID-19 test. While there was not a significant difference as observed in most other postoperative complications, extra care should be paid to preoperative cardiac and pulmonary optimization to mitigate the risks of anesthesia. A positive COVID-19 diagnosis alone is not a contraindication to ankle fracture surgery; however, waiting 2 weeks after an infection, which may be undertaken while also splinting and elevating the extremity to reduce swelling, may reduce mortality.

## Supplemental Material

sj-docx-1-fas-10.1177_19386400241274539 – Supplemental material for Navigating Ankle Fracture Surgery in the Shadow of COVID-19 Supplemental material, sj-docx-1-fas-10.1177_19386400241274539 for Navigating Ankle Fracture Surgery in the Shadow of COVID-19 by Justin P. Chan, Henry Hoang, Amanda Anderson and Andrew R. Hsu in Foot & Ankle Specialist
