# Supplementary material for: Navigating Ankle Fracture Surgery in the Shadow of COVID-19
Source: Foot Ankle Spec. 2024 Sep 20;19(2):187–95. doi: 10.1177/19386400241274539 (PMC12957410; doi:10.1177/19386400241274539)
Supplement: sj-docx-1-fas-10.1177_19386400241274539 – Supplemental material for Navigating Ankle Fracture Surgery in the Shadow of COVID-19 [file sj-docx-1-fas-10.1177_19386400241274539.docx]

| DVT | I82.401, I82.402, I82.403, I82.409, I82.49, I82.491, I82.492, I82.493, I82.499, I82.4Y, I82.4Y1, I82.4Y2, I82.4Y3, I82.4Y9, I82.4Z, I82.4Z1, I82.4Z2, I82.4Z3, I82.4Z9, I82.62, I82.621, I82.622, I82.623, I82.629, I26, I26.0, I26.01, I26.09, I26.90, I26.93, I26.99 |
| --- | --- |
| Sepsis | B37.7, B37.7, B37.700, B37.7, A41.802,  A41.101, A41.504, A41.807, A26.7, A26.7,  A26.7, A26.700, A54.86, A41.50, A41.805,  A32.7, A32.7, A32.7, A32.700, A41.59, A41,  A41, A41, A41.800, A41.8, A41.8, A41.8,  A41.89, A40.8, A40.800, A40.8, A40.8,  O98.801, A41.505, O85, 670.2, O85.x00,  O85, O85, 670.22, 670.24, 670.2, A02.100,  A02.1, A02.1, A02.1, 995.91, T81.411,  A41.4, A41.400, A41.4, A41.4, A41.81,  A41.51, A41.3, A41.300, A41.3, A41.3,  A41.02, A41.01, A41.5, A41.500, A41.5,  A41.5, A41.100, A41.1, A41.1, A41.1,  A41.52, A41.53, A41.000, A41.0, A41.0,  A41.0, A40.0, A40.0, A40.000, A40.0,  A40.1, A40.1, A40.100, A40.1, A40.200,  A40.2, A40.2, A40.3, A40.3, A40.300,  A40.3, A41.2, A41.2, A41.200, A41.2,  O86.04, T81.44, T81.44XA, T81.44XS,  T81.44XD, O03.87, O08.82, O07.37, O03.37,  O04.87, A41.901, P36.500, P36.5, P36.5,  P36.5, P36.4, P36.4, P36.4, P36.400, P36.3,  P36.300, P36.3, P36.3, P36.1, P36.1,  P36.100, P36.1, P36.39, P36.19, P36.200,  P36.2, P36.2, P36.2, P36.0, P36.0, P36.000,  P36.0, P36.30, P36.10, A41.9, A41.9,  A41.900, A41.9, 771.81, 995.92, R65.2,  R65.20, R65.21, P36.301, P36.101, A40,  A40, A40, A40.9, A40.9, A40.900, A40.9,  P36.000, P36.0, P36.30, P36.10, A41.9,  A41.9, A41.900, A41.9, 771.81, 995.92,  R65.2, R65.20, R65.21, P36.301, P36.101,  A40, A40, A40, A40.9, A40.9, A40.900,  A40.9 |
| Surgical Site Infection | T81.42, T81.42XA, T81.42XS, T81.42XD, D998.3, D998.31, D998.32, D998.33, T81.3XA, T81.3XD, T81.3XS, T81.31XA, T81.31XD, T81.31XS, T81.32XA, T81.32XD, T81.32XS, T81.33XA, T81.33XD, T81.33XS |
| Bleeding | 998.11, 998.12, 998.13, D78.01, D78.02, D78.21, D78.22, E36.01, E36.02, E89.810, E89.811, G97.31, G97.32, G97.51, G97.52, H59.111, H59.112, H59.113, H59.119, H59.121, H59.122, H59.123, H59.129, H59.311, H59.312, H59.313, H59.319, H59.321, H59.322, H59.323, H59.329, H95.21, H95.22, H95.41, H95.42, I97.410, I97.411, I97.418, I97.42, I97.610, I97.611, I97.618, I97.620, J95.61, J95.62, J95.830, J95.831, K91.61, K91.62, K91.840, K91.841, L76.01, L76.02, L76.21, L76.22, M96.810, M96.811, M96.830, M96.831, N99.61, N99.62, N99.820, N99.821, T888XXA |
| Acute Kidney Injury | N17, N14, R39.2, D59.3, k76.7, t79.5, r39.2, 90.4, n99.0, n17.0, n17.1, n17.2, n17.8, n17.9, n19, 584, 584.5, 584.6, 584.7, 584.8, 584.9 |

Supplementary Table 1: ICD10 codes for Post-Operative Complications
